# Four-dimensional velocity encoded magnetic resonance imaging for more accurate blood flow quantification in complex flow

**DOI:** 10.1186/1532-429X-13-S1-P225

**Published:** 2011-02-02

**Authors:** Sarah Nordmeyer, Eugénie Riesenkampff, Felix Berger, Titus Kuehne

**Affiliations:** 1 Deutsches Herzzentrum Berlin, Berlin, Germany

## Purpose

Valvar stenosis causes accelerated and often complex non-laminar flow patterns that are sometimes difficult to assess quantitatively using 2D VEC MRI. We sought to evaluate the use of 4D VEC MRI for measuring flow velocities and volumes in complex flow conditions.

## Materials and methods

Maximal flow velocities (Vmax) and flow volumes (SV) were quantified by 2D and 4D VEC MRI in healthy volunteers (n=7) and patients with aortic (n=7) or pulmonary valve stenosis (n=10). Measurements were performed immediately above the aortic or pulmonary valve (=level 1). Using 4D VEC MRI additional measurements were performed at 3 further levels in the ascending aorta (levels 2-4) and at 2 in the MPA (levels 2-3). In each patient flow volumes measured in the non-pathologic MPA or Aorta served as internal control for expected flow volumes in the absence of shunt flow.

## Results

**Figure 1:**
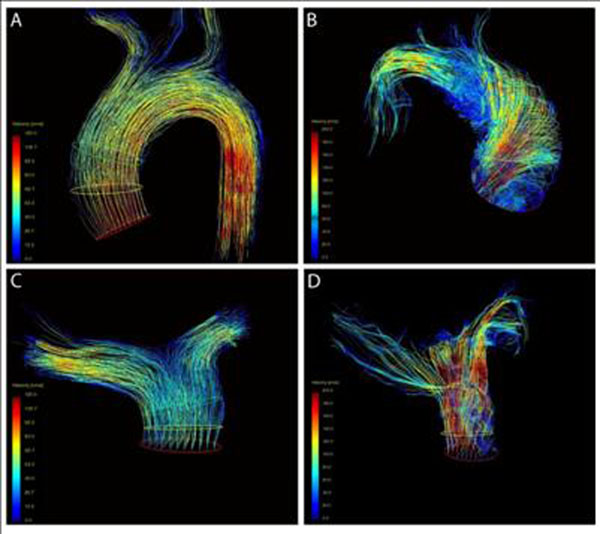


2D and 4D VEC MRI render highly comparable stroke volumes in healthy volunteers and patients when measured at the same anatomical region. In healthy volunteers flow patterns along the ascending aorta and main, right and left pulmonary artery were laminar in part swirling but not turbulent (Figure 1 A,C). SV and Vmax at different levels in the ascending aorta and different levels in the pulmonary trunk were comparable (2D vs 4D VEC MRI). In patients with aortic or pulmonary valve stenosis flow patterns showed a large variance with vortex formation, helical blood flow and presumably local turbulences (Figure 1 B,D). As a result, Vmax were found at different locations of the vessel that were often missed when using 2D or 4D VEC MRI at level 1. In concordance, there were significant differences in Vmax between measurements performed in level 1-4. On the other hand, in patients we noted a significant larger variance in SV and Vmax than in healthy volunteers that is presumably caused by local turbulences.

## Conclusion

4D VEC MRI, like Doppler echocardiography, can target and thus improve the assessment of peak flow velocity in valvar stenosis. In addition, 4D flow allows visualizing regions with very complex flow that might be associated with turbulences. Therefore, measurement errors can be potentially avoided.

